# Effect of Eplontersen in Patients With Hereditary Transthyretin Amyloidosis With Polyneuropathy Across Genetic Variants: An Exploratory Analysis From the NEURO‐TTRansform Trial

**DOI:** 10.1111/ene.70580

**Published:** 2026-03-30

**Authors:** Julian D. Gillmore, David Adams, Markus Weiler, Ahmad Masri, Laura Obici, Jonatan Nåtman, T. Jesse Kwoh, Barry Reicher, James Revkin, Márcia Waddington Cruz, Morie Gertz, Chi‐Chao Chao

**Affiliations:** ^1^ National Amyloidosis Centre University College London London UK; ^2^ Neurology Department, CHU de Bicêtre, AP‐HP Université Paris‐Saclay Le‐Kremlin‐Bicêtre France; ^3^ Amyloidosis Center and Department of Neurology Heidelberg University Hospital Heidelberg Germany; ^4^ Oregon Health & Science University Portland Oregon USA; ^5^ Amyloidosis Research and Treatment Centre IRCCS Fondazione Policlinico San Matteo Pavia Italy; ^6^ BioPharmaceuticals Business Unit AstraZeneca Gothenburg Sweden; ^7^ Clinical Development Ionis Pharmaceuticals, Inc. Carlsbad California USA; ^8^ Late‐Stage Development, Cardiovascular, Renal, and Metabolism (CVRM) BioPharmaceuticals R&D, AstraZeneca Gaithersburg Maryland USA; ^9^ CEPARM, Amyloidosis Center University Hospital, Federal University of Rio de Janeiro Rio de Janeiro Brazil; ^10^ Department of Hematology Mayo Clinic Rochester Minnesota USA; ^11^ Department of Neurology National Taiwan University Hospital Taipei Taiwan

**Keywords:** ATTR amyloidosis, ATTRv‐PN, eplontersen, polyneuropathy, quality of life, Val30Met

## Abstract

**Background:**

This exploratory analysis of the NEURO‐TTRansform Phase 3 trial evaluated the efficacy of eplontersen in patients with hereditary transthyretin (ATTRv) amyloidosis with polyneuropathy by genetic variant.

**Methods:**

Changes from baseline in NEURO‐TTRansform primary endpoints serum transthyretin (TTR) at Week 65, modified Neuropathy Impairment Score+7 (mNIS+7) composite score, and Norfolk Quality of Life‐Diabetic Neuropathy (Norfolk QoL‐DN) total score at Week 66 were evaluated in patients with early‐onset (aged < 50 years) and late‐onset (aged ≥ 50 years) Val30Met (p.Val50Met) or non‐Val30Met ATTRv amyloidosis with polyneuropathy. Secondary endpoints from NEURO‐TTRansform were also evaluated by genetic variant.

**Results:**

In total, 144 patients with early‐onset (*n =* 54) or late‐onset (*n =* 31) Val30Met or non‐Val30Met (*n =* 59), ATTRv amyloidosis with polyneuropathy were randomized to eplontersen. A further 60 patients from NEURO‐TTR with early‐onset (*n =* 16) or late‐onset (*n =* 17) Val30Met or non‐Val30Met (*n =* 27), served as a historical placebo group. The mean percentage difference (95% confidence interval) in serum TTR was −79.9 (−87.8, −72.0), −85.0 (−93.3, −76.6), and −70.6 (−77.7, −63.5) with eplontersen versus placebo in the early‐ and late‐onset Val30Met, and non‐Val30Met groups, respectively. Across subgroups, the change from baseline to Week 66 in mNIS+7 composite score was generally well maintained, and the Norfolk QoL‐DN total score improved with eplontersen versus worsening with placebo. The Polyneuropathy Disability score was maintained in most patients. From baseline to Week 65, the modified body mass index was maintained with eplontersen compared to a marked reduction (worsening) for placebo.

**Conclusions:**

Findings were suggestive of consistent benefits in reducing neuropathy impairment and improving QoL with eplontersen versus historical placebo, across *TTR* variants.

**Trial Registration:**

ClinicalTrials.gov: NCT04136184, NCT01737398

## Graphical abstract

In this exploratory analysis of the NEURO‐TTRansform Phase 3 trial, the efficacy of eplontersen was evaluated in patients with early‐ or late‐onset Val30Met (p.Val50Met) or non‐Val30Met hereditary transthyretin amyloidosis with polyneuropathy. Eplontersen halted neuropathy impairment as measured by the mNIS+7 composite score and improved Norfolk QoL‐DN total score from baseline to Week 66 versus historical placebo. In summary, findings suggestive of consistent benefits in reducing neuropathy impairment and improving quality of life were observed with eplontersen versus historical placebo, regardless of the *TTR* variant.

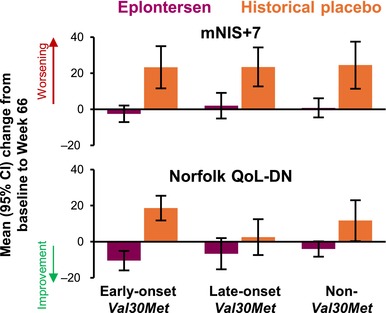

## 1 Introduction

Hereditary transthyretin (ATTRv) amyloidosis is a progressive and fatal autosomal dominant disorder caused by point mutations in the transthyretin (*TTR*) gene [1, 2]. Abnormal TTR protein accumulates as amyloid deposits in the peripheral and autonomic nerves and other organs, resulting in peripheral and autonomic neuropathy and other manifestations including cardiomyopathy [1].

The clinical presentation of ATTRv amyloidosis is heterogeneous, often with a mixed phenotype of neurologic and cardiomyopathic manifestations due to its multisystemic nature; certain pathogenic variants may be associated with a predominantly neurologic or a predominantly cardiac phenotype [1]. Val30Met (p.Val50Met) is the most commonly identified neuropathic *TTR* variant in ATTRv amyloidosis globally and can manifest as early‐onset (< 50 years old) or late‐onset (≥ 50 years old) disease [3, 4]. Most patients with early‐onset Val30Met disease have a predominantly neurologic phenotype without amyloid cardiomyopathy as opposed to patients with late‐onset Val30Met disease who typically have a mixed polyneuropathy and cardiomyopathy phenotype [3, 4].

Another frequently identified *TTR* variant, Val122Ile (p.Val142Ile), is associated with a predominantly cardiomyopathic phenotype causing heart failure, although patients may also experience symptoms of polyneuropathy [5, 6]. The Val122Ile variant is found in 3%–4% of the African American population [7]. Other *TTR* variants, including Thr60Ala (p.Thr80Ala) and Ala97Ser (p.Ala117Ser), are associated with a mixed phenotype. Thr60Ala affects 1% of the population in North‐West Ireland [8], and a study by the UK National Amyloidosis Centre reported cardiac involvement in nearly all patients with the Thr60Ala variant who were referred with neuropathy [9]. Ala97Ser is the most frequently detected *TTR* variant in Taiwanese patients and is associated with a mixed phenotype with late‐onset polyneuropathy and cardiomyopathy [10].

The recent approval of disease‐modifying therapies for the treatment of ATTRv amyloidosis with polyneuropathy, together with the availability of genetic testing, has highlighted the importance of timely diagnosis and treatment in generating a meaningful impact on health and quality of life (QoL) outcomes for patients [1, 11]. Current standard‐of‐care therapies for ATTRv amyloidosis include TTR stabilizers and *TTR* gene silencers [11].

Eplontersen is an antisense oligonucleotide that is approved in multiple countries including the United States, Canada, and the European Union for use in adults with ATTRv amyloidosis with polyneuropathy [12, 13, 14]. In the NEURO‐TTRansform Phase 3 trial, eplontersen significantly lowered serum TTR concentration through 65 weeks and halted neuropathy impairment while improving QoL versus a historical placebo through 66 weeks in adult patients with ATTRv amyloidosis with polyneuropathy [15].

Given the heterogeneity in clinical presentation and underlying genotypes associated with ATTRv amyloidosis, it is important to establish the consistency of treatment effect of therapies for ATTRv amyloidosis with polyneuropathy in diverse patient populations. This exploratory analysis aimed to evaluate the efficacy of eplontersen versus a historical placebo in patients with either early‐onset (aged < 50 years) or late‐onset (aged ≥ 50 years) Val30Met or non‐Val30Met ATTRv amyloidosis with polyneuropathy.

## 2 Method

### 2.1 Trial Design

NEURO‐TTRansform was a global, multicenter, open‐label, Phase 3 trial conducted from December 2019 through April 2023. The trial protocol and amendments were approved for each participating center by the relevant local institutional review boards or ethics committees [16, 17]. NEURO‐TTRansform was conducted in accordance with the International Council for Harmonization guidelines, and written informed consent was provided by all patients prior to enrollment.

Detailed inclusion and exclusion criteria have been reported previously [15, 17]. Briefly, adults aged 18–82 years with a diagnosis of ATTRv amyloidosis with polyneuropathy Coutinho Stage 1 (ambulatory without assistance) or 2 (ambulatory with assistance) were eligible for enrollment if they had a Neuropathy Impairment Score between 10 and 130 points and a documented *TTR* sequence variant.

Patients were randomly assigned 6:1 to open‐label treatment with subcutaneous eplontersen (45 mg every 4 weeks) through Week 81 or inotersen (300 mg weekly) from Weeks 1 to 34 with a planned switch to eplontersen 45 mg every 4 weeks for study Weeks 37–81 [15, 18]. The switch group was not included in the current analysis. A historical placebo group was derived from the randomized NEURO‐TTR trial [16]. Eligibility criteria and endpoints were consistent between the NEURO‐TTRansform and NEURO‐TTR trials [16].

### 2.2 Exploratory Analysis

This exploratory analysis included all participants who were randomized to eplontersen in NEURO‐TTRansform and received ≥ 1 dose of eplontersen, and participants who were randomized to placebo in NEURO‐TTR.

Patients with ATTRv amyloidosis with polyneuropathy were grouped into three subgroups according to the *TTR* variant: early‐onset (aged < 50 years) Val30Met; late‐onset (aged ≥ 50 years) Val30Met; and non‐Val30Met.

### 2.3 NEURO‐TTRansform Primary Endpoints

Endpoints from NEURO‐TTRansform have previously been described in detail [15, 17].

The mean percentage change in serum TTR concentration (g/L) from baseline to Week 65 for the eplontersen and placebo groups was reported by *TTR* variant subgroup.

Disease progression and QoL were assessed using validated instruments. Changes from baseline to Week 66 were measured for the modified Neuropathy Impairment Score + 7 (mNIS+7) composite score (range, −22.3 to 346.3) and the Norfolk Quality of Life‐Diabetic Neuropathy (QoL‐DN) total score (range, −4 to 136). Higher scores indicate worsening neuropathy and poorer QoL, respectively, while a decrease in score indicates improvement.

### 2.4 NEURO‐TTRansform Secondary Endpoints

Changes from baseline to Week 66 were measured for the Neuropathy Symptom and Change (NSC) total score, and from baseline to Week 65 for the Physical Component Summary (PCS) score of the 36‐Item Short‐Form Health Survey (SF‐36), and the Polyneuropathy Disability (PND) score. Higher NSC total scores indicate worsening, and increasing PCS scores indicate improvement.

On the PND scale, a score of I indicates sensory disturbance but with preserved walking capacity; II indicates unassisted walking but with difficulty; IIIa indicates one stick or crutch is required for walking; IIIb indicates two sticks or crutches are required for walking; and IV indicates the patient is wheelchair‐bound or bedridden.

Change from baseline to Week 65 in modified body mass index (mBMI), calculated as BMI × albumin g/L, served as a measure of nutritional status and was adjusted for low serum albumin levels.

### 2.5 Statistical Analysis

Baseline patient demographic and disease characteristics were summarized descriptively. The mNIS+7, Norfolk QoL‐DN, and NSC analyses included participants with both baseline and Week 66 values. The serum TTR, PCS, mBMI, and PND analyses included participants with both baseline and Week 65 values. Analyses were descriptive with no formal statistical tests performed. Multiple endpoints were examined without adjustment for multiplicity.

### 2.6 Sensitivity Analyses

To ensure the robustness of the primary findings, two sensitivity analyses were performed. The first excluded patients with the Ala97Ser variant. The second was a propensity score weighted analysis. Propensity scores were estimated using a logistic regression model with treatment assignment (eplontersen vs. historical placebo) as the dependent variable and the following baseline covariates as predictors: disease stage, baseline mNIS+7 score, prior treatment, age, disease duration, and transthyretin amyloid cardiomyopathy diagnosis (ATTR‐CM). ATTR‐CM diagnosis was excluded from the model for the early‐onset Val30Met subgroup, as no patients had the diagnosis. Further details on the propensity score weighted analysis are given in the Supporting Informations.

## 3 Results

### 3.1 Baseline Patient Characteristics

This analysis included 144 patients with early‐onset (*n =* 54) or late‐onset (*n =* 31) Val30Met, or non‐Val30Met (*n =* 59), ATTRv amyloidosis with polyneuropathy who were randomized to receive eplontersen. A further 60 patients with early‐onset (*n =* 16) or late‐onset (*n =* 17) Val30Met, or non‐Val30Met (*n =* 27), ATTRv amyloidosis with polyneuropathy served as a historical placebo group.

Across both the early‐onset and late‐onset Val30Met variant subgroups, more than two‐thirds of patients were male, with a slightly lower proportion of males in the non‐Val30Met subgroups (62.7% and 59.3% for the eplontersen and placebo groups, respectively; Table 1).

**TABLE 1:** Baseline characteristics by *TTR* variant.

Characteristic	Early‐onset Val30Met (aged < 50 years)		Late‐onset Val30Met (aged ≥ 50 years)		Non‐Val30Met	
	Eplontersen (*n =* 54)	Historical placebo^a^ (*n =* 16)	Eplontersen (*n =* 31)	Historical placebo^a^ (*n =* 17)	Eplontersen (*n =* 59)	Historical placebo^a^ (*n =* 27)
Age (years), mean (SD)	39.2 (6.3)	40.1 (6.3)	68.2 (6.1)	69.1 (7.3)	57.7 (13.3)	65.0 (8.3)
Male, *n* (%)	41 (75.9)	11 (68.8)	22 (71.0)	14 (82.4)	37 (62.7)	16 (59.3)
Race, *n* (%)						
White	51 (94.4)	15 (93.8)	28 (93.3)	15 (88.2)	33 (55.9)	23 (85.2)
Asian	0 (0.0)	0 (0.0)	0 (0.0)	1 (5.9)	22 (37.3)	2 (7.4)
Black or African American	1 (1.9)	0 (0.0)	0 (0.0)	0 (0.0)	4 (6.8)	1 (3.7)
Other or multiple	2 (3.7)	1 (6.2)	2 (6.7)	1 (5.9)	0 (0.0)	1 (3.7)
Missing	0 (0.0)	0 (0.0)	1 (3.2)	0 (0.0)	0 (0.0)	0 (0.0)
Geographic region, *n* (%)						
North America	2 (3.7)	0 (0.0)	2 (6.5)	7 (41.2)	17 (28.8)	19 (70.4)
Central and Southern America	27 (50.0)	8 (50.0)	12 (38.7)	2 (11.8)	7 (11.9)	1 (3.7)
Europe, Australia, and New Zealand	25 (46.3)	8 (50.0)	17 (54.8)	8 (47.1)	14 (23.7)	7 (25.9)
Taiwan	0 (0.0)	0 (0.0)	0 (0.0)	0 (0.0)	21 (35.6)	0 (0.0)
Body weight (kg), mean (SD)	66.9 (14.0)	65.2 (15.7)	73.3 (13.1)	71.1 (10.4)	71.8 (18.0)	74.5 (22.5)
*n*	52	16	29	17	57	27
BMI (kg/m^2^), mean (SD)	22.5 (4.7)	22.2 (4.9)	26.0 (4.1)	24.2 (2.6)	25.2 (5.0)	25.4 (5.6)
*n*	52	16	29	17	57	27
mBMI, mean (SD)	967.2 (249.6)	968.6 (231.8)	1072.5 (187.6)	1036.6 (143.4)	1055.5 (236.0)	1106.4 (259.3)
Albumin (g/L), mean (SD)	42.8 (3.2)	44.2 (3.5)	41.6 (2.2)	42.9 (2.9)	42.0 (2.9)	43.4 (2.9)
Median duration of disease from diagnosis of ATTRv amyloidosis with polyneuropathy, months (IQR)	53.0 (30.0; 90.0)	65.0 (34.5; 100.0)	20.0 (7.5; 55.0)	21.0 (7.0; 32.0)	17.0 (4.0; 35.5)	9.0 (5.0; 44.5)
Median duration of disease from onset of symptoms of ATTRv amyloidosis with polyneuropathy, months (IQR)	73.5 (43.8; 102.0)	64.0 (47.0; 88.2)	42.0 (28.5; 69.0)	39.0 (27.0; 54.0)	48.5 (23.5; 84.0)	48.0 (23.5; 104.0)
Cardiomyopathy, *n* (%)	0 (0.0)	0 (0.0)	14 (45.2)	7 (41.2)	25 (42.4)	15 (55.6)
PND score, *n* (%)						
I	20 (37.0)	3 (18.8)	9 (29.0)	5 (29.4)	27 (46.6)	15 (55.6)
II	29 (53.7)	11 (68.8)	14 (45.2)	3 (17.6)	18 (31.0)	5 (18.5)
IIIa	4 (7.4)	2 (12.5)	3 (9.7)	7 (41.2)	9 (15.5)	6 (22.2)
IIIb	1 (1.9)	0 (0.0)	5 (16.1)	2 (11.8)	4 (6.9)	1 (3.7)
Previous treatment with tafamidis or diflunisal, *n* (%)	45 (83.3)	11 (68.8)	22 (71.0)	9 (52.9)	33 (55.9)	16 (59.3)
mNIS + 7 composite score, mean (SD)^b^	83.1 (42.9)	77.6 (46.8)	91.6 (39.3)	86.5 (37.0)	74.2 (45.3)	65.7 (34.1)
*n*	54	16	30	17	53	26
Norfolk QoL‐DN total score, mean (SD)^b^	40.8 (25.7)	47.6 (22.8)	50.8 (23.7)	52.4 (26.8)	43.6 (28.8)	46.9 (29.6)
NSC total score, mean (SD)	26.6 (11.0)	26.0 (14.1)	24.1 (11.6)	23.3 (12.2)	19.5 (13.2)	21.0 (12.0)
PCS of SF‐36 score, mean (SD)	41.3 (8.7)	39.1 (11.6)	36.8 (6.4)	34.1 (8.5)	39.7 (10.7)	38.1 (9.4)
ATTRv amyloidosis with polyneuropathy stage, *n* (%)						
Stage 1	50 (92.6)	14 (87.5)	21 (67.7)	8 (47.1)	44 (74.6)	20 (74.1)
Stage 2	4 (7.4)	2 (12.5)	10 (32.3)	9 (52.9)	15 (25.4)	7 (25.9)

Abbreviations: ATTRv, hereditary transthyretin; BMI, body mass index; IQR, interquartile range; mBMI, modified body mass index; mNIS+7, modified Neuropathy Impairment Score + 7; Norfolk QoL‐DN, Norfolk Quality of Life‐Diabetic Neuropathy; NSC, Neuropathy Symptom and Change; PCS of SF‐36, Physical Component Summary of Short Form‐36 questionnaire; PND, Polyneuropathy Disability; SD, standard deviation; TTR, transthyretin.

^a^ From the NEURO‐TTR study.

^b^ Higher scores indicate a worse health state.

At baseline, the mean age ranged from 57.7 to 69.1 years in the late‐onset Val30Met and non‐Val30Met subgroups, compared with 39.2–40.1 years in the early‐onset Val30Met subgroup. The mean age at baseline was broadly balanced between patients receiving eplontersen and placebo within the *TTR* variant subgroups.

In the early‐onset and late‐onset Val30Met subgroups, 88.2%–94.4% of patients were White. In contrast, in the non‐Val30Met subgroup, 55.9% and 85.2% of patients were White in the eplontersen‐ and placebo‐treated groups, respectively. This is reflected in the greater proportion of patients from Taiwan in the eplontersen‐treated non‐Val30Met subgroup (35.6% compared with 0% in all other groups). Correspondingly, 21 patients in the eplontersen‐treated non‐Val30Met subgroup had the Ala97Ser variant, compared with 1 patient in the placebo group.

The median duration of disease from the diagnosis of ATTRv amyloidosis with polyneuropathy was > 30 months longer for patients in the early‐onset Val30Met subgroup, and duration of disease from the onset of symptoms was also longer when compared with patients in the other *TTR* variant subgroups.

Most patients had Stage 1 ATTRv amyloidosis with polyneuropathy, except for those in the late‐onset Val30Met placebo group, where there was a greater proportion of patients with a PND score of IIIa (41.2%) compared with other subgroups (range, 7.4%–22.4%).

Among patients with Val30Met ATTRv amyloidosis with polyneuropathy, 45.2% and 41.2% of those with late‐onset disease treated with eplontersen and placebo, respectively, had a clinical diagnosis of cardiomyopathy (reported as yes/no in the electronic data capture) at baseline; no patients with early‐onset Val30Met had cardiomyopathy. Clinical cardiomyopathy was diagnosed in 42.4% versus 55.6% of eplontersen‐ and placebo‐treated patients, respectively, in the non‐Val30Met variant subgroup.

At baseline, mNIS+7 scores and Norfolk scores were highest in the late‐onset Val30Met subgroup, and generally lower in the other early‐onset Val30Met and non‐Val30Met variant subgroups.

### 3.2 Serum TTR Concentration

**FIGURE 1:**
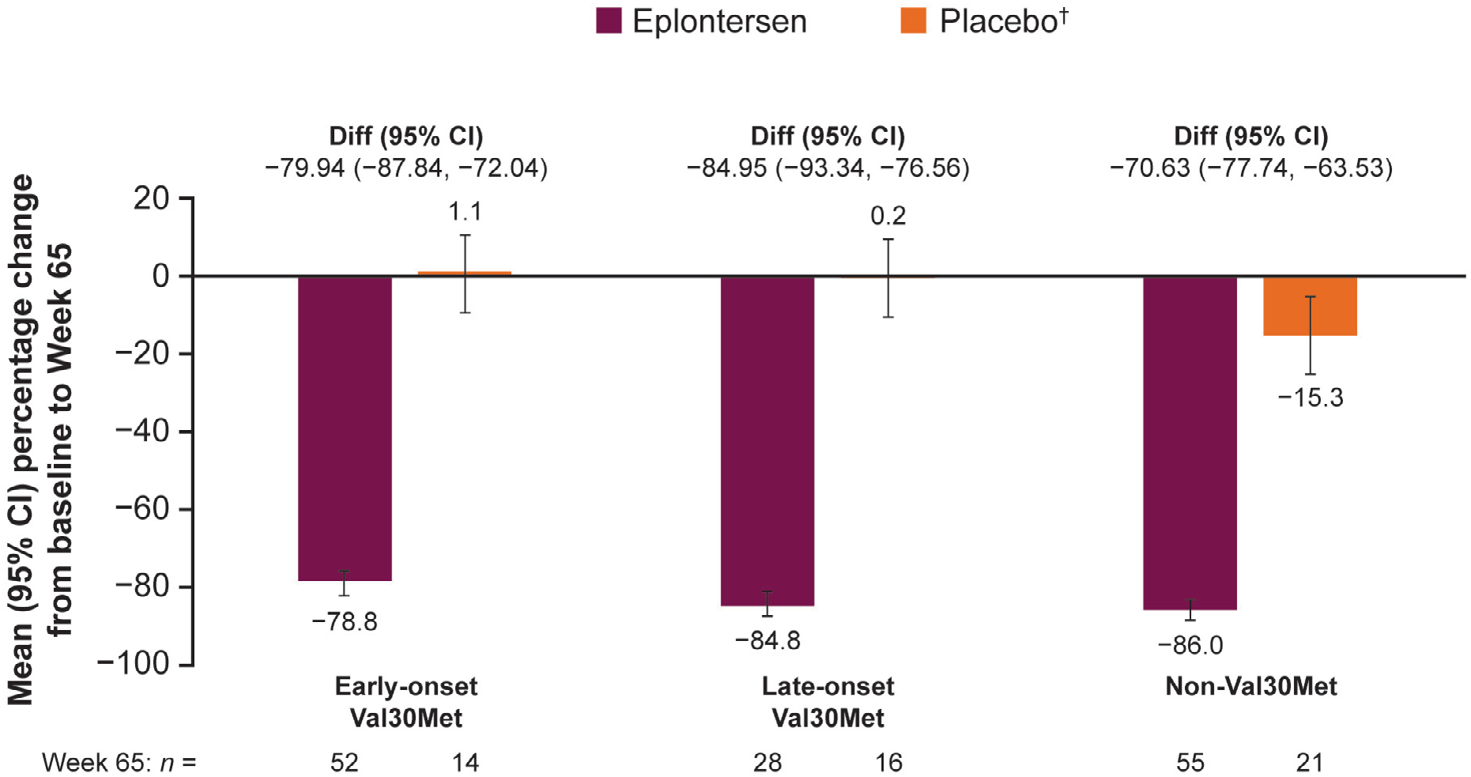
Mean (95% CI) percentage change from baseline to Week 65 in serum TTR concentration by *TTR* variant. ^†^From the NEURO‐TTR study. CI, confidence interval; Diff., difference in means; TTR, transthyretin.

From baseline to Week 65, there was a consistent reduction of approximately 70%–85% in serum TTR concentration with eplontersen across all *TTR* variant subgroups, versus no change with placebo in the Val30Met subgroups and a reduction of 15% in the non‐Val30Met subgroup (Figure 1).

### 3.3 Neuropathy Impairment and Patient QoL

**FIGURE 2:**
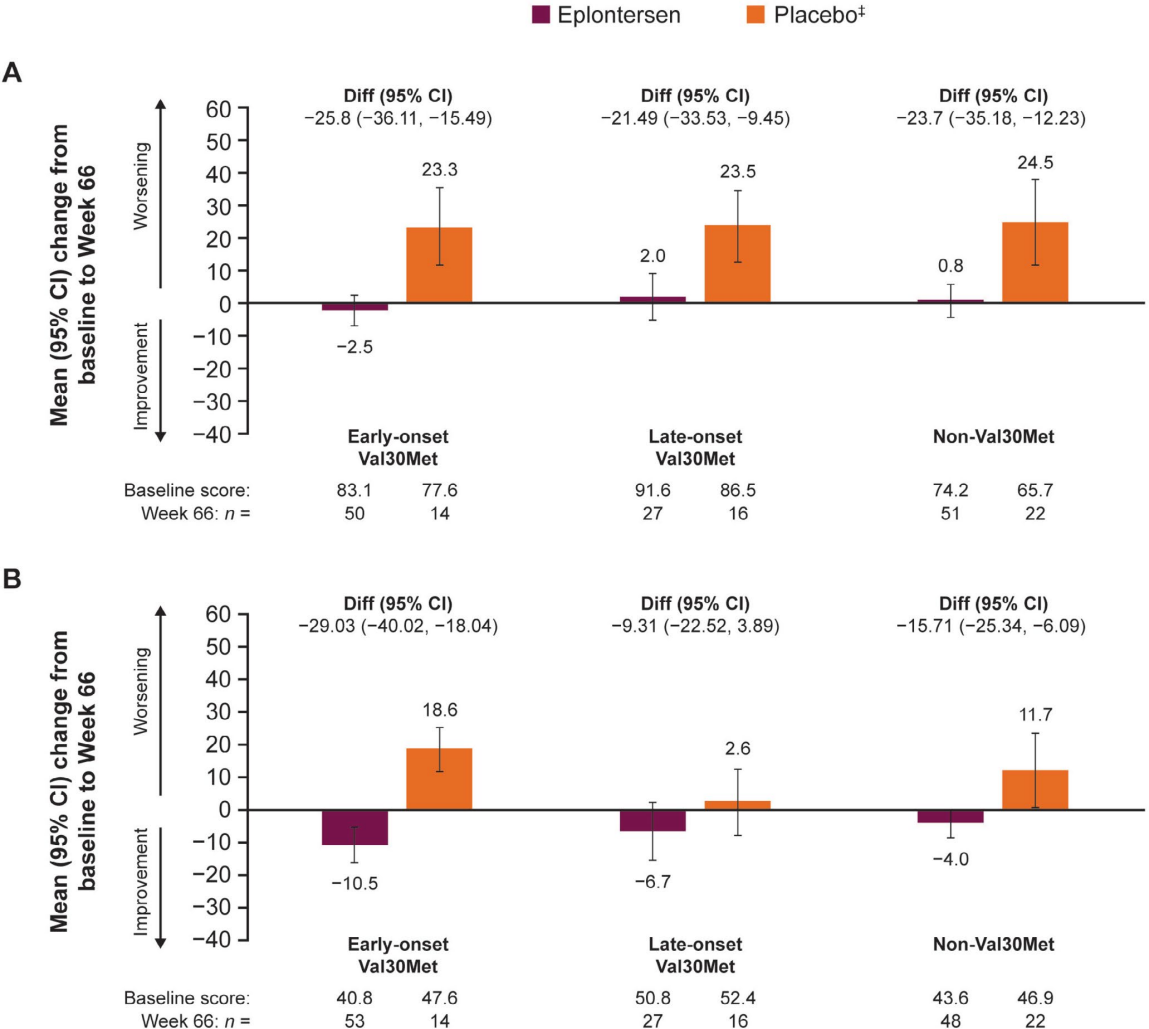
Mean (95% CI) change from baseline to Week 66 in (A) mNIS+7 composite score (higher scores indicate a worse health state) and (B) Norfolk QoL‐DN total score by *TTR* variant (higher scores indicate a worse health state). ^‡^From the NEURO‐TTR study. CI, confidence interval; Diff., difference in means; mNIS+7, modified Neuropathy Impairment Score + 7; Norfolk QoL‐DN, Norfolk Quality of Life‐Diabetic Neuropathy; TTR, transthyretin.

Across all *TTR* variant subgroups, neuropathy impairment, as measured by the mean change from baseline to Week 66 in mNIS+7 composite score, did not worsen with eplontersen compared with substantial worsening with placebo (Figure 2A). Norfolk QoL‐DN total score improved in all *TTR* variant subgroups with eplontersen versus placebo; the greatest improvement was observed for the early‐onset Val30Met subgroup (mean difference [95% confidence interval; CI]: −29.0 [−40.0, −18.0]) compared with the late‐onset Val30Met (−9.3 [−22.5, 3.9]) and non‐Val30Met (−15.7 [−25.3, −6.1]) subgroups (Figure 2B).

### 3.4 NSC, PCS of SF‐36, and mBMI

**FIGURE 3:**
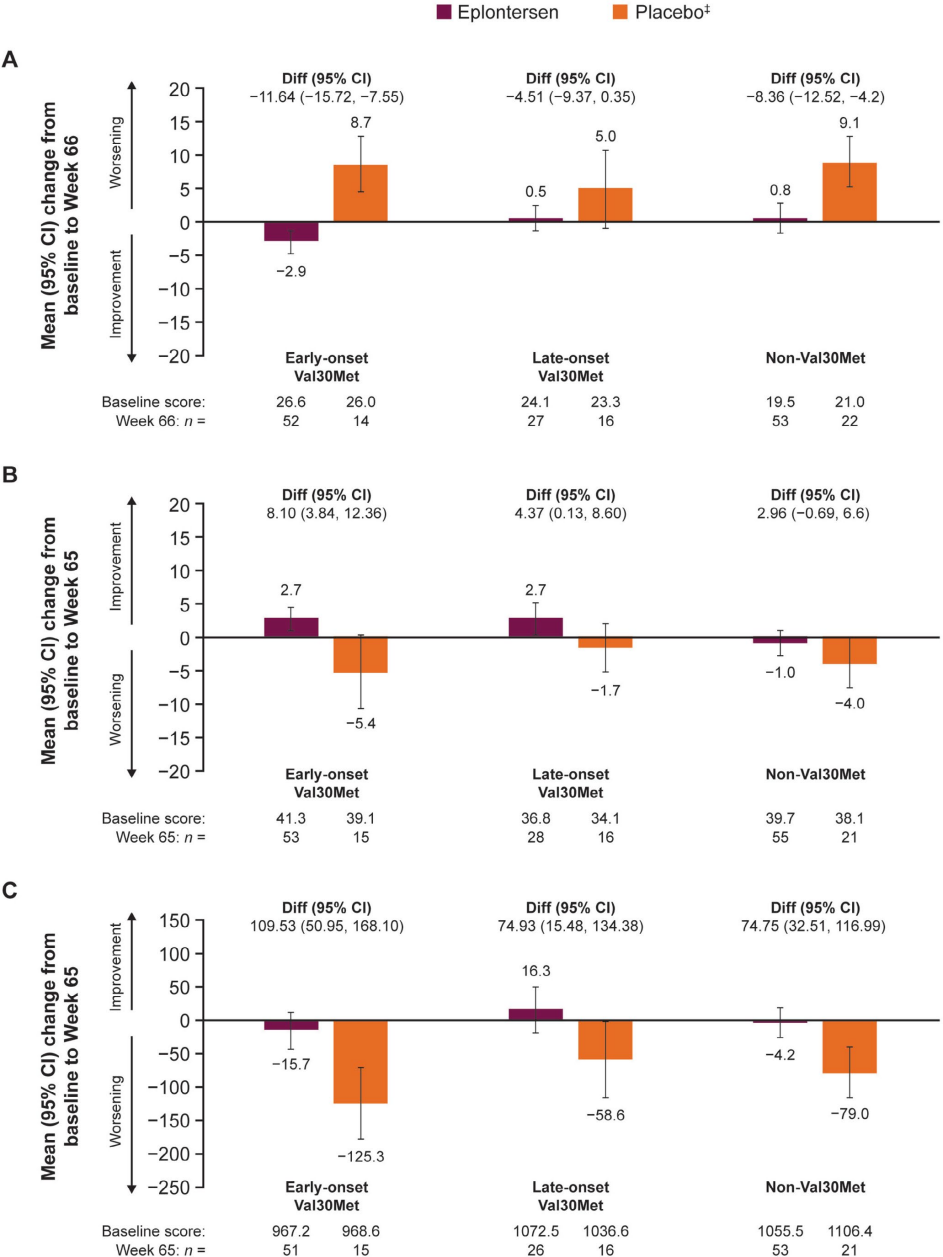
Change from baseline to Week 65/66 in (A) NSC (higher scores indicate a worse health state), (B) PCS of SF‐36, and (C) mBMI score by *TTR* variant. ^‡^From the NEURO‐TTR study. Diff., difference in means; mBMI, modified body mass index; NSC, Neuropathy Symptom and Change; PCS of SF‐36, Physical Component Summary of Short Form‐36 questionnaire; TTR, transthyretin.

Across all *TTR* variant subgroups, the mean change from baseline to Week 66 in the NSC score was generally maintained with eplontersen, with a slight improvement observed in those in the early‐onset Val30Met subgroup, but worsened with placebo across all *TTR* variant subgroups. Mean changes from baseline to Week 65 in PCS of SF‐36 scores were modestly in favor of eplontersen compared with placebo (Figure 3A,B).

Nutritional status, as measured by changes in mBMI from baseline to Week 65, remained generally stable with eplontersen compared with a reduction (worsening) with placebo, which was most pronounced in the early‐onset Val30Met subgroup (mean difference [95% CI]: 109.5 [51.0, 168.1]) compared with the late‐onset Val30Met (74.9 [15.5, 134.4]) and non‐Val30Met (74.8 [32.5, 117.0]) subgroups (Figure 3C).

Results from a sensitivity analysis excluding 21 Taiwanese patients in the eplontersen group and one North American patient in the placebo group with the Ala97Ser variant were consistent with the main analysis across all outcomes examined (Figure S1). A propensity score weighted analysis, adjusting for disease stage, baseline mNIS+7 score, prior treatment, age, disease duration, and ATTR‐CM diagnosis, was conducted. Propensity score weighting improved covariate balance relative to the unadjusted comparison in the overall cohort and across subgroups (Tables S1–S3). Results of the propensity score weighted analysis were consistent with the unweighted analysis (Figure S1).

### 3.5 PND Score

**FIGURE 4:**
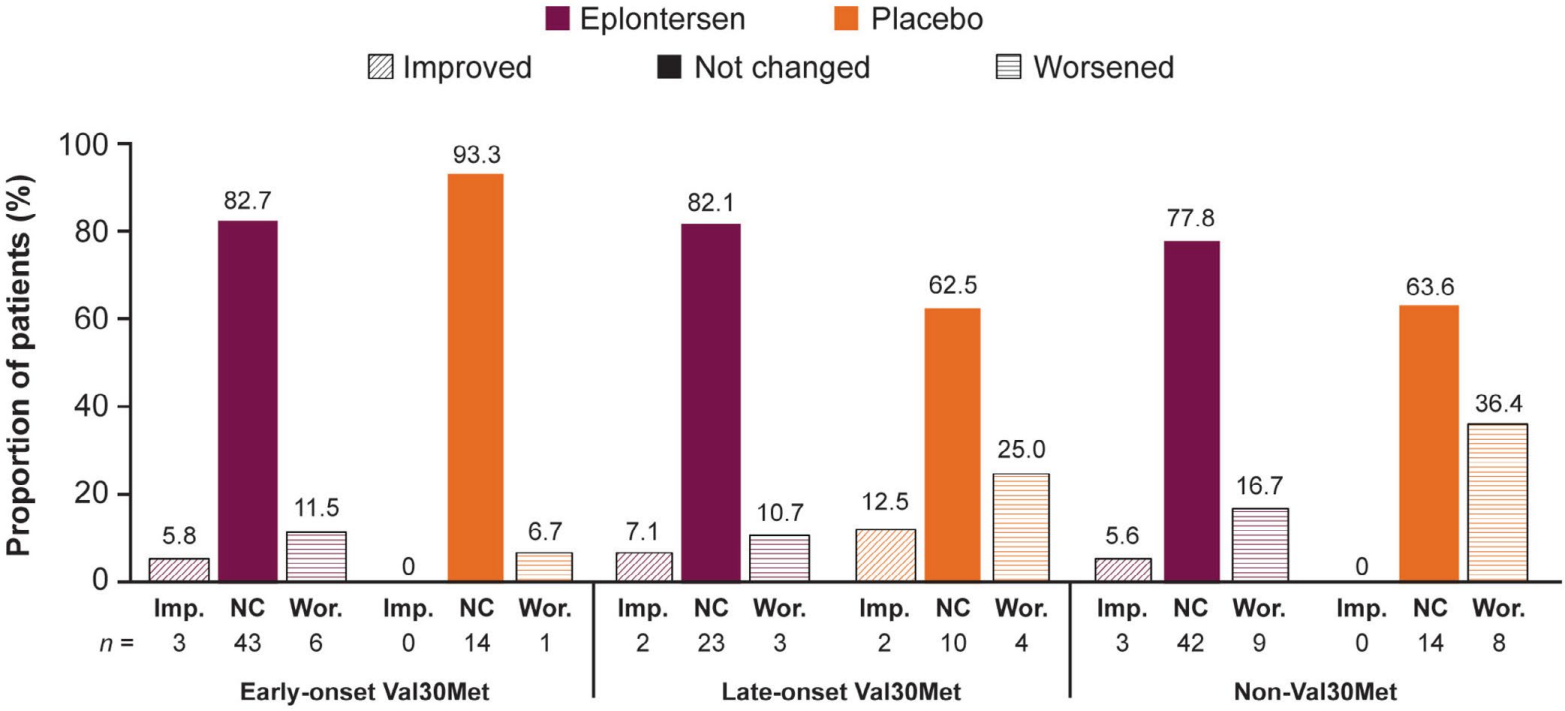
Change in PND score at Week 65 by *TTR* variant. Imp., improved; NC, not changed; PND, Polyneuropathy Disability; TTR, transthyretin; Wor., worsened.

In the late‐onset Val30Met and non‐Val30Met subgroups, the proportion of patients who experienced worsening in PND score was approximately half with eplontersen versus placebo (10.7% vs. 25.0% and 16.7% vs. 36.4%). In the early‐onset Val30Met subgroup, the proportion of patients with worsening in PND score was similar with eplontersen (11.5%) and placebo (6.7%), and the proportion that improved was 5.8% versus 0%, respectively. Overall, most patients were unchanged in their PND score (62.5%–93.3%), and the number of patients experiencing an improvement was 0%–12.5% across all *TTR* variant subgroups and treatment groups (Figure 4).

## 4 Discussion

In this exploratory analysis of the Phase 3 NEURO‐TTRansform trial, eplontersen halted the progression of neuropathy impairment and improved QoL in patients with ATTRv amyloidosis with polyneuropathy compared with placebo; these effects were numerically consistent across the *TTR* variants and phenotypes analyzed. Mean serum TTR concentration was reduced by approximately 80% from baseline with eplontersen across all *TTR* variant subgroups, compared with no change with placebo in the Val30Met subgroups and a reduction of 15% in the non‐Val30Met subgroup. Changes in the mNIS+7 and Norfolk QoL‐DN total score demonstrated that eplontersen halted the deterioration of neuropathy symptoms and improved QoL versus placebo. Nutritional status, as measured by mBMI, was maintained with eplontersen whereas it worsened with placebo. The deterioration of walking ability, as shown by PND scores, was stopped or markedly reduced in patients with late‐onset Val30Met ATTRv amyloidosis with polyneuropathy, who typically present with large fiber neuropathy [19, 20]. The benefits of eplontersen compared with placebo were apparent in all *TTR* variant subgroups examined in this analysis and were consistent regardless of genotype.

ATTRv amyloidosis is a heterogeneous disease both in terms of the underlying *TTR* variant and its clinical presentation. Over 130 *TTR* variants have been identified to date, with genotype–phenotype correlations partially explaining the differences in disease manifestation between patients [11]. Consistent with previous studies that reported greater neurologic impairment in patients with late‐ versus early‐onset Val30Met ATTRv amyloidosis with polyneuropathy, patients in this study with late‐onset Val30Met had higher (worse) mNIS+7 scores at baseline compared with early‐onset Val30Met [3]. Moreover, in the current study, there was a trend toward patients in the early‐onset Val30Met subgroup experiencing improvement in neuropathy symptoms and QoL as measured by the mNIS+7 composite score, Norfolk QoL‐DN total score, and NSC scores compared with patients with late‐onset Val30Met ATTRv amyloidosis. Prior studies have reported that patients with late‐onset Val30Met ATTRv amyloidosis experience more severe and faster‐progressing neuropathic disease compared with patients with early‐onset Val30Met ATTRv amyloidosis [3, 21].

In this study, the change in nutritional status, as measured by mBMI, was maintained in patients receiving eplontersen, while mBMI worsened in patients receiving placebo. For patients treated with eplontersen, those in the late‐onset Val30Met subgroup had a numerical improvement in mBMI, compared with patients in the early‐onset Val30Met subgroup who experienced a slight worsening. Patients with early‐onset disease experience more gastrointestinal (GI) symptoms than patients with late‐onset disease, and patients with Val30Met experience more GI symptoms than those who are non‐Val30Met [22]. The prevalence of GI symptoms, including nutritional status, in ATTRv amyloidosis is associated with the duration of the disease [22]. In our study, patients with early‐onset Val30Met had a longer duration of disease compared with the other *TTR* variant subgroups and may therefore have been more affected by GI manifestations. Nevertheless, eplontersen treatment maintained nutritional status across all *TTR* variant subgroups compared with placebo.

Such differences in disease manifestations and prognosis between *TTR* variants highlight the importance of assessing the efficacy of eplontersen in a patient cohort that reflects the real‐world *TTR* genotype diversity. The composition of *TTR* variants that make up the patient population of major Phase 3 trials of *TTR* silencers largely reflects the overall ATTRv amyloidosis with polyneuropathy population [15, 23, 24]. Overall, in NEURO‐TTRansform, 60% of patients had the Val30Met variant, while 14%, 3%, and 2% had the Ala97Ser, Thr60Ala, and Val122Ile variants, respectively [23]. This proportion of patients with the Val30Met variant is approaching that reported in the global THAOS study, which identified that 73% of patients with a *TTR* gene variant had the Val30Met variant [25]. Furthermore, the distribution of *TTR* variants in NEURO‐TTRansform varied by geographic location, reflecting the diversity in the global distribution of these variants, with a larger representation of the Val122Ile variant in North America [15, 23, 26].

Currently, there is a lack of evidence directly comparing the efficacy of therapies in patients with ATTRv amyloidosis with polyneuropathy caused by different *TTR* variants. Prior studies have suggested variability in the efficacy of TTR stabilizers in patients with different *TTR* variants and phenotypes [27, 28]. The TTR stabilizer tafamidis can delay peripheral neurologic impairment in patients with early‐stage Val30Met ATTRv amyloidosis [29, 30]; however, some studies have shown progression of neuropathy symptoms in patients with advanced‐stage late‐onset Val30Met ATTRv amyloidosis and non‐Val30Met ATTRv amyloidosis [27, 28].

Limitations of this analysis include the relatively small sample sizes of the subgroups, and the follow‐up duration (65/66 weeks) may be insufficient to capture long‐term variant‐specific differences in treatment response. Several subgroup analyses include fewer than 20 patients. This limits precision, as reflected in the wide 95% confidence intervals, and constrains the interpretability of between‐group differences. An imbalance in the number of patients in the eplontersen and placebo treatment groups precluded the analysis of the Ala97Ser, Thr60Ala, and Val122Ile variants individually; this may have resulted in masking of the effects of individual variants. For ethical reasons, the efficacy of eplontersen in the NEURO‐TTRansform trial was compared with a historical placebo group from the previously conducted NEURO‐TTR trial. The implications of the use of a historical placebo group in NEURO‐TTRansform have been discussed previously [31]. Some baseline imbalances were noted due to the introduction of temporal and population differences, including a higher mean age in the historical placebo versus eplontersen groups. This was particularly evident in the distribution of the Ala97Ser variant between the eplontersen and historical placebo groups, where the inclusion of Taiwan as a study location in the NEURO‐TTRansform trial, but not the NEURO‐TTR trial, resulted in an imbalance of patients with the Ala97Ser variant between treatment groups. These imbalances may confound comparisons between groups. Results of a sensitivity analysis excluding patients with the Ala97Ser variant, and a propensity score weighted analysis adjusting for disease stage, prior treatment, age, and disease duration, were consistent with the main analysis. Further research from studies, including real‐world studies, of a larger sample size and longer duration would be valuable to enable the detection of differences between variant groups and be more generalizable to the population. Lastly, the proportion of patients with cardiomyopathy may be underestimated as sensitive imaging was not performed. These limitations, along with the exploratory design and post hoc nature of this analysis, suggest that the findings should be considered hypothesis‐generating, rather than confirmatory.

Given the progressive nature of ATTRv amyloidosis, timely diagnosis is important for early initiation of effective treatment that may enhance QoL for patients. Eplontersen has demonstrated numerically consistent and significant benefits versus historical placebo in reducing neuropathy impairment, improving QoL, and in maintaining nutritional status in patients with ATTRv amyloidosis with polyneuropathy across *TTR* genetic variants. These results underscore the importance of early diagnosis and treatment, and affirm the broad applicability of eplontersen in a diverse patient population.

## Author Contributions

**Julian D. Gillmore:** conceptualization, investigation, resources, writing – original draft, methodology, validation, writing – review and editing. **David Adams:** conceptualization, investigation, resources, writing – original draft, methodology, validation, writing – review and editing. **Markus Weiler:** conceptualization, investigation, resources, writing – original draft, methodology, validation, writing – review and editing. **Ahmad Masri:** conceptualization, investigation, resources, writing – original draft, methodology, validation, writing – review and editing. **Laura Obici:** conceptualization, investigation, resources, writing – original draft, methodology, validation, writing – review and editing. **Jonatan Nåtman:** conceptualization, investigation, resources, writing – original draft, methodology, validation, writing – review and editing. **T. Jesse Kwoh:** conceptualization, investigation, resources, writing – original draft, methodology, validation, writing – review and editing. **Barry Reicher:** conceptualization, investigation, resources, writing – original draft, methodology, validation, writing – review and editing. **James Revkin:** conceptualization, investigation, resources, writing – original draft, methodology, validation, writing – review and editing. **Márcia Waddington Cruz:** conceptualization, investigation, resources, writing – original draft, methodology, validation, writing – review and editing. **Morie Gertz:** conceptualization, investigation, resources, writing – original draft, methodology, validation, writing – review and editing. **Chi‐Chao Chao:** conceptualization, investigation, resources, writing – original draft, methodology, validation, writing – review and editing.

## Funding

The NEURO‐TTRansform trial was sponsored by Ionis Pharmaceuticals Inc. The analyses presented here were supported by AstraZeneca.

## Conflicts of Interest

J.D.G. is a consultant for Alnylam, Intellia, AstraZeneca, Attralus, Ionis, BridgeBio, Lycia, Bayer, and Pfizer. D.A. is a consultant for AstraZeneca, Alnylam, BridgeBio, and Intellia. M.W. is a consultant for Akcea, Alnylam, Biogen, Hoffmann‐La Roche, Novartis, Novo Nordisk, Pfizer, and Sobi; has received speaker fees from Akcea, Alnylam, AstraZeneca, and Biogen; and has received financial support for conference attendance from Akcea, Alnylam, AstraZeneca, Ionis, and Pfizer; and is a member of the European Reference Network for Neuromuscular Diseases (ERN EURO‐NMD). A.M. has received research funding from Pfizer, Ionis, Attralus, and Cytokinetics; is a consultant to Cytokinetics, BMS, Eidos/BridgeBio, Pfizer, Ionis, Lexicon, Attralus, Alnylam, Haya, Alexion, Akros, Prothena, BioMarin, AstraZeneca, and Tenaya. L.O. is a consultant to, and/or has received speaker fees from, Alnylam, Intellia, AstraZeneca, Ionis, BridgeBio, Novo Nordisk, SOBI, and Pfizer. T.J.K. was an employee and shareholder of Ionis during the conduct of the studies and is presently a contractor and shareholder of Ionis. B.R., J.R., and J.N. are employees of, and hold stock in, AstraZeneca. M.W.C. is a principal investigator for the NEURO‐TTRansform trial and is a consultant to Ionis. M.G. has reported receiving personal fees from Ionis, Alnylam, Prothena, Janssen, Sanofi, Juno, Physicians Education Resource, Johnson & Johnson, Celgene, and Research to Practice; serves on the data and safety monitoring board for AbbVie; receives research funding from Aptitude Health; receives meeting fees from Ashfield and Sorrento; and develops educational materials for i3Health. C.‐C.C. has no conflicts of interest to declare.

## Supporting information


**Appendix S1:** Methods.
**Table S1:** Covariate balance table for the propensity score analysis for the early‐onset Val30Met (aged < 50 years) group.
**Table S2:** Covariate balance table for the propensity score analysis for the late‐onset Val30Met (aged ≥ 50 years) group.
**Table S3:** Covariate balance table for the propensity score analysis for the non‐Val30Met group.
**Figure S1:** Sensitivity analysis for study endpoints excluding patients with the Ala97Ser variant and propensity score weighted analysis^†^ by *TTR* variant.

## Data Availability

Data requests from qualified researchers will be considered once all three of the following criteria are met: (1) 12 months from marketing approval of the study drug in both the United States and the European Union; (2) 18 months from the conclusion of the study; and (3) 6 months from the publication of this article. For additional information, visit https://vivli.org/ourmembers/ionis.
