# Supplementary material for: Effect of Eplontersen in Patients With Hereditary Transthyretin Amyloidosis With Polyneuropathy Across Genetic Variants: An Exploratory Analysis From the NEURO‐TTRansform Trial
Source: Eur J Neurol. 2026 Mar 30;33(4):e70580. doi: 10.1111/ene.70580 (PMC13140992; doi:10.1111/ene.70580)
Supplement: Supplementary file 1 — Appendix S1: Methods. Table S1: Covariate balance table for the propensity score analysis for the early‐onset Val30Met (aged < 50 years) group. Table S2: Covariate balance table for the propensity score analysis for the late‐onset Val30Met (aged ≥ 50 years) group. Table S3: Covariate balance table for the propensity score analysis for the non‐Val30Met group. Figure S1: Sensitivity analysis for study endpoints excluding patients with the Ala97Ser variant and propensity score weighted analysis† by TTR variant. [file ENE-33-e70580-s001.docx]

# Supplementary Materials

## Propensity Score Weighted Analysis

### Supplementary Methods

Propensity scores were estimated using a logistic regression model with treatment assignment (eplontersen vs historical placebo) as the dependent variable and disease stage, baseline mNIS+7 score, prior treatment, age disease duration and transthyretin amyloid cardiomyopathy diagnosis (ATTR-CM) as covariates. ATTR-CM diagnosis was excluded from the model for the early-onset Val30Met subgroup, as no patients had the diagnosis. Models were fitted in the overall population and separately within each subgroup to allow subgroup-specific confounding structures. To estimate the treatment effect among treated patients, average-treatment-effect-on-the-treated weights were applied; weight 1 was assigned to eplontersen-treated patients and weight p/(1−p) to historical placebo patients, where p denotes the estimated propensity score. Covariate balance was quantified before and after weighting using absolute standardized mean differences (SMDs). SMDs <0.1 were considered as indicative of good balance and SMDs <0.2 as acceptable balance. The distribution of weights was assessed.

TABLE S1. Covariate balance table for the propensity score analysis for the early-onset Val30Met (aged <50 years) group

| **Characteristic** | **Before weighting** | | | **After weighting** | | |
| --- | --- | --- | --- | --- | --- | --- |
|  | **Eplontersen (*n =* 54)** | **Historical placebo^a^ (*n =* 16)** | **SMD** | **Eplontersen (*n =* 54)** | **Historical placebo^a^ (*n =* 16)** | **SMD** |
| **Age (years), mean (SD)** | 39.2 (6.3) | 40.1 (6.3) | 0.13 | 39.2 (6.3) | 39.1 (5.6) | 0.02 |
| **Female, n (%)** | 13.0 (24.1) | 5.0 (31.2) | 0.16 | 13.0 (24.1) | 2.6 (16.5) | 0.19 |
| **Geographic region, *n* (%)** |  |  | 0.28 |  |  | 0.5 |
| North America | 2.0 (3.7) | 0.0 (0.0) |  | 2.0 (3.7) | 0.0 (0.0) |  |
| South America | 27.0 (50.0) | 8.0 (50.0) |  | 27.0 (50.0) | 5.2 (32.2) |  |
| Europe | 25.0 (46.3) | 8.0 (50.0) |  | 25.0 (46.3) | 10.8 (67.8) |  |
| **Duration of disease from onset of symptoms of ATTRv amyloidosis with polyneuropathy, months, mean (SD)** | 76.6 (40.5) | 66.8 (31.2) | 0.27 | 76.6 (40.5) | 74.6 (28.1) | 0.06 |
| **Cardiomyopathy, *n* (%)** | 54.0 (100.0) | 16.0 (100.0) | <0.001 | 54.0 (100.0) | 16.0 (100.0) | <0.001 |
| **mNIS+7 composite score, mean (SD)^b^** | 83.1 (43.0) | 77.6 (46.8) | 0.12 | 83.1 (43.0) | 85.8 (42.7) | 0.06 |
| **Previous treatment with tafamidis or diflunisal, *n* (%)** | 45.0 (83.3) | 11.0 (68.8) | 0.35 | 45.0 (83.3) | 13.7 (85.6) | 0.06 |
| **ATTRv amyloidosis with polyneuropathy stage 2, *n* (%)** | 4.0 (7.4) | 2.0 (12.5) | 0.17 | 4.0 (7.4) | 1.1 (7.0) | 0.01 |

Abbreviations: ATTRv, hereditary transthyretin; mNIS+7, modified Neuropathy Impairment Score +7; SD, standard deviation; SMD, standardized mean difference..

^a^From the NEURO-TTR study.

^b^Higher scores indicate a worse health state.

TABLE S2. Covariate balance table for the propensity score analysis for the late-onset Val30Met (aged ≥50 years) group

| **Characteristic** | **Before weighting** | | | **After weighting** | | |
| --- | --- | --- | --- | --- | --- | --- |
|  | **Eplontersen (*n =* 31)** | **Historical placebo^a^ (*n =* 17)** | **SMD** | **Eplontersen (*n =* 31)** | **Historical placebo^a^ (*n =* 17)** | **SMD** |
| **Age (years), mean (SD)** | 68.2 (6.1) | 69.1 (7.3) | 0.12 | 68.23 (6.1) | 67.6 (7.6) | 0.09 |
| **Female, n (%)** | 9.0 (29.0) | 3.0 (17.6) | 0.27 | 9.0 (29.0) | 2.7 (15.6) | 0.33 |
| **Geographic region, *n* (%)** |  |  | 1.01 |  |  | 1.05 |
| North America | 2.0 ( 6.5) | 7.0 (41.2) |  | 2.0 ( 6.5) | 6.7 (39.5) |  |
| South America | 12.0 (38.7) | 2.0 (11.8) |  | 12.0 (38.7) | 1.5 ( 8.6) |  |
| Europe | 17.0 (54.8) | 8.0 (47.1) |  | 17.0 (54.8) | 8.8 (51.9) |  |
| **Duration of disease from onset of symptoms of ATTRv amyloidosis with polyneuropathy, months, mean (SD)** | 57.84 (48.5) | 41.9 (22.1) | 0.42 | 57.8 (48.5) | 43.8 (21.5) | 0.38 |
| **Cardiomyopathy, *n* (%)** | 14.0 (45.2) | 7.0 (41.2) | 0.08 | 14.0 (45.2) | 8.4 (49.4) | 0.08 |
| **mNIS+7 composite score, mean (SD)^b^** | 91.6 (39.3) | 86.5 (37.0) | 0.13 | 91.6 (39.3) | 85.6 (33.1) | 0.17 |
| **Previous treatment with tafamidis or diflunisal, *n* (%)** | 22.0 (71.0) | 9.0 (52.9) | 0.38 | 22.0 (71.0) | 11.7 (68.6) | 0.05 |
| **ATTRv amyloidosis with polyneuropathy stage 2, *n* (%)** | 10.0 (32.3) | 9.0 (52.9) | 0.43 | 10.0 (32.3) | 5.8 (34.3) | 0.04 |

Abbreviations: ATTRv, hereditary transthyretin; mNIS+7, modified Neuropathy Impairment Score +7; SD, standard deviation; SMD, standardized mean difference.

^a^From the NEURO-TTR study.

^b^Higher scores indicate a worse health state.

TABLE S3. Covariate balance table for the propensity score analysis for the non-Val30Met group

| **Characteristic** | **Before weighting** | | | **After weighting** | | |
| --- | --- | --- | --- | --- | --- | --- |
|  | **Eplontersen (*n =* 59)** | **Historical placebo^a^ (*n =* 27)** | **SMD** | **Eplontersen (*n =* 59)** | **Historical placebo^a^ (*n =* 27)** | **SMD** |
| **Age (years), mean (SD)** | 57.7 (13.3) | 65.0 (8.3) | 0.67 | 57.5 (13.3) | 59.4 (11.3) | 0.15 |
| **Female, n (%)** | 22.0 (37.3) | 11.0 (40.7) | 0.07 | 22.0 (37.9) | 9.7 (35.8) | 0.04 |
| **Geographic region, *n* (%)** |  |  | 1.33 |  |  | 1.37 |
| North America | 17.0 (28.8) | 19.0 (70.4) |  | 17.0 (29.3) | 19.1 (70.8) |  |
| South America | 7.0 (11.9) | 1.0 ( 3.7) |  | 7.0 (12.1) | 0.5 ( 1.8) |  |
| Europe | 12.0 (20.3) | 7.0 (25.9) |  | 12.0 (20.7) | 7.4 (27.3) |  |
| Rest of World | 23.0 (39.0) | 0.0 ( 0.0) |  | 22.0 (37.9) | 0.0 ( 0.0) |  |
| **Duration of disease from onset of symptoms of ATTRv amyloidosis with polyneuropathy, months, mean (SD)** | 64.8 (59.6) | 76.3 (69.8) | 0.18 | 64.8 (59.6) | 57.5 (55.3) | 0.13 |
| **Cardiomyopathy, *n* (%)** | 25.0 (42.4) | 15.0 (55.6) | 0.27 | 25.0 (43.1) | 9.4 (34.7) | 0.17 |
| **mNIS+7 composite score, mean (SD)^b^** | 74.2 (45.3) | 65.7 (34.1) | 0.21 | 73.9 (45.6) | 68.5 (33.7) | 0.14 |
| **Previous treatment with tafamidis or diflunisal, *n* (%)** | 33.0 (55.9) | 16.0 (59.3) | 0.07 | 33.0 (56.9) | 11.7 (43.2) | 0.28 |
| **ATTRv amyloidosis with polyneuropathy stage 2, *n* (%)** | 15.0 (25.4) | 7.0 (25.9) | 0.01 | 15.0 (25.9) | 7.1 (26.1) | 0.01 |

Abbreviations: ATTRv, hereditary transthyretin; mNIS+7, modified Neuropathy Impairment Score +7; SD, standard deviation; SMD, standardized mean difference.

^a^From the NEURO-TTR study.

^b^Higher scores indicate a worse health state.

FIGURE S1. Sensitivity analysis for study endpoints excluding patients with the Ala97Ser variant and propensity score weighted analysis^†^ by *TTR* variant.


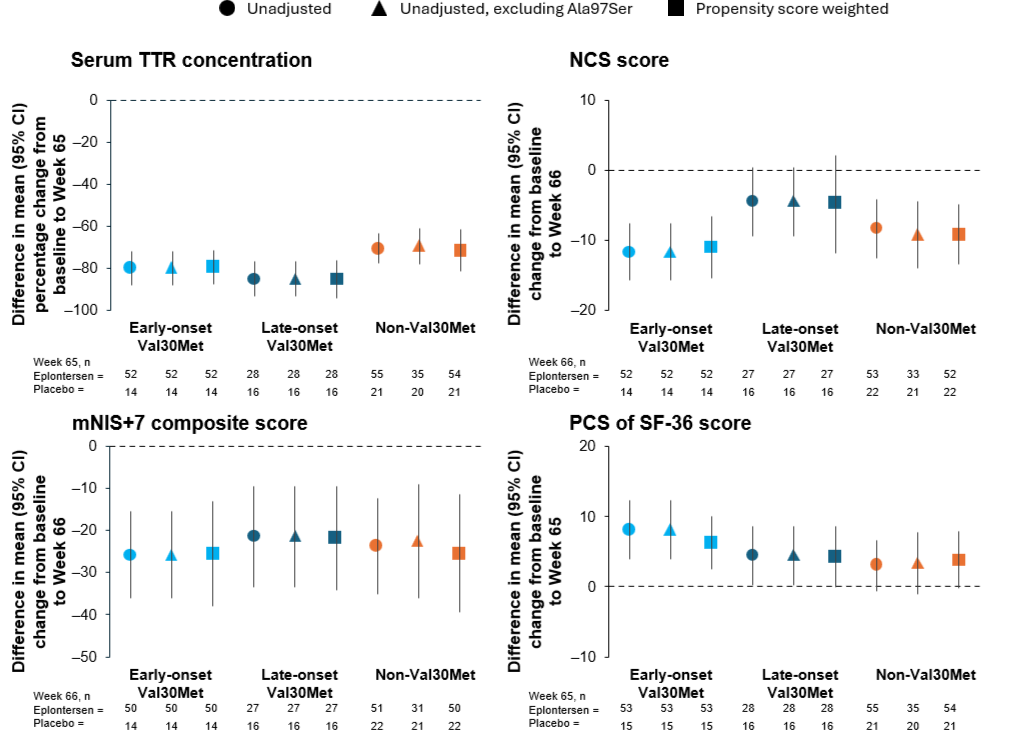


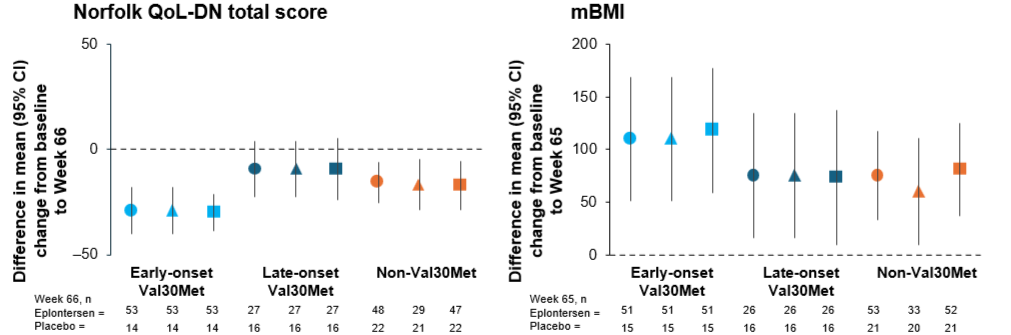


^†^Propensity score weighted analysis was adjusted for disease stage, baseline mNIS+7 score, prior treatment, age, disease duration, and transthyretin amyloid cardiomyopathy diagnosis. Patients in the placebo arm were weighted to resemble the covariate distribution in the eplontersen arm. Propensity score weighting improved covariate balance relative to the unadjusted comparison in the overall cohort and across subgroups (most post-weighting SMDs <0.2). In the late-onset Val30Met subgroup, subgroup, disease duration and sex remained unbalanced with SMDs of 0.38 and 0.35, respectively. In the non-Val30Met subgroup, previous treatment was unbalanced with an SMD of 0.28 post weighting. Weight distributions were stable overall; the maximum weight was 5.97.
BMI, modified body mass index; mNIS+7, modified Neuropathy Impairment Score +7; Norfolk QoL-DN, Norfolk Quality of Life-Diabetic Neuropathy; NSC, Neuropathy Symptom and Change; PCS of SF-36, Physical Component Summary of Short Form-36 questionnaire; SMD, standardized mean differences; TTR, transthyretin.

Treatment with Eplontersen in Patients With Hereditary Transthyretin Amyloidosis With Polyneuropathy With Different Genetic Variants

**AI Plain Language Summary**

**Why was this research done?**

Hereditary transthyretin (ATTRv) amyloidosis is a genetic disorder caused by buildup of abnormal transthyretin (TTR) proteins, causing damage to nerves and other organs. This research aimed to explore how well the drug eplontersen works in treating patients with ATTRv amyloidosis with nerve damage (polyneuropathy) who have differences in their TTR gene (also known as genetic variants). One common genetic variant is called Val30Met, and can cause symptoms to start at a younger (under 50 years) or older (50 years or older) age. Understanding the effects of eplontersen across various genetic variants of ATTRv amyloidosis can help improve treatment strategies and patient outcomes.

**How was this research done?**
The research was conducted through the NEURO-TTRansform Phase 3 trial, a global study from December 2019 to April 2023. Researchers analyzed data from 144 patients treated with eplontersen and compared their results to 60 patients who received placebo in a previous trial. Patients were divided into subgroups based on their genetic variant: early-onset Val30Met, late-onset Val30Met, and non-Val30Met.

**What were the results of this research and what does this mean for patients?**

The study found that eplontersen consistently reduced serum TTR levels by approximately 70%-85% across all genetic variant groups. It also halted the worsening of nerve damage and improved patients' quality of life compared to placebo. Nutritional status (the body’s ability to take in and use nutrients), measured by modified body mass index (mBMI), was maintained with eplontersen but worsened with placebo. The findings suggest that eplontersen is effective in treating ATTRv amyloidosis with polyneuropathy across different genetic variants. This underscores the importance of early diagnosis and treatment of the disease to improve patient outcomes. The broad applicability of eplontersen means it could be a valuable treatment option for a diverse patient population suffering from this genetic disorder.

**Who funded this study?**

The NEURO-TTRansform trial was sponsored by Ionis Pharmaceuticals, Inc. The analyses presented here were supported by AstraZeneca.

AI-assisted technology was used to support the development of this plain language summary.

**Where can I access more information?**

Data described in this plain language summary may be obtained in accordance with AstraZeneca’s data sharing policy described [here](https://www.astrazenecaclinicaltrials.com/our-transparency-commitments/).
